# A Unique Case of Infected Laryngeal Tophi With Underlying Gout

**DOI:** 10.7759/cureus.74363

**Published:** 2024-11-24

**Authors:** Muhammad Hazeem Akashah, Siti Hajar Sanudin, Charlene P Malakun

**Affiliations:** 1 Otolaryngology - Head and Neck Surgery, Hospital Queen Elizabeth, Kota Kinabalu, MYS

**Keywords:** head and neck region, larynx, monosodium urate crystals, purine, tophaceous gout

## Abstract

Gout is a disorder of purine metabolism described by the deposition of monosodium urate crystals with rare involvement in the head and neck. This is the first laryngeal gout case reported in Sabah, Malaysia. A 50-year-old gentleman with a long history of gouty arthritis presented with acute painless anterior neck swelling for two weeks. An ultrasound of the neck was done, which showed a midline neck mass with intralesional calcification of the cartilaginous component with suspicious erosion of the thyroid cartilage, which was suggestive of thyroid chondroma. However, a computed tomography (CT) scan of the neck showed the fluid collection in the subperichondrium of anterior thyroid cartilage with amorphous calcification, which raised suspicion of infected laryngeal gout. Based on histology and intraoperative findings, laryngeal gout was diagnosed as noted multiple tophy crystals. Microscopic examination confirmed birefringent crystalline deposits. The pathophysiology and management of this rare clinical entity are discussed. We reported this case due to its rarity as well as to increase awareness of laryngeal gout.

## Introduction

Laryngeal involvement in gout, leading to the formation of tophi, is exceedingly rare. When complicated by infection, management becomes challenging due to the delicate nature of the laryngeal structures and the potential for airway compromise. We present a unique case of a 50-year-old gentleman presented with a sudden onset of painless anterior neck swelling for 2 weeks with no prior history of trauma or fever. He had hoarseness and dysphagia. Investigations were done followed by surgery. For this work to be published, the patient’s written agreement and approval were acquired.

## Case presentation

**Figure 1:**
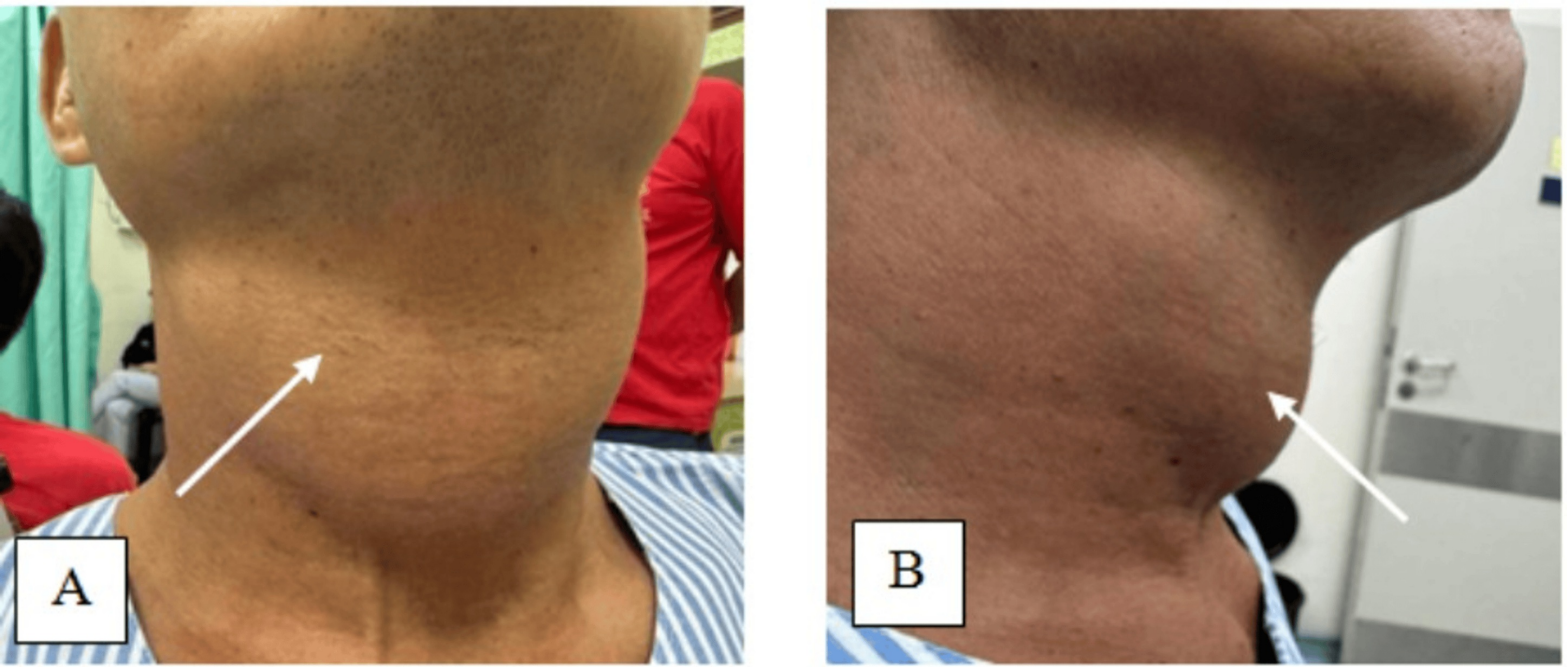
Anterior (A) and lateral view (B) of neck swelling confined to the level of thyroid cartilage area (white arrow)

**Figure 2:**
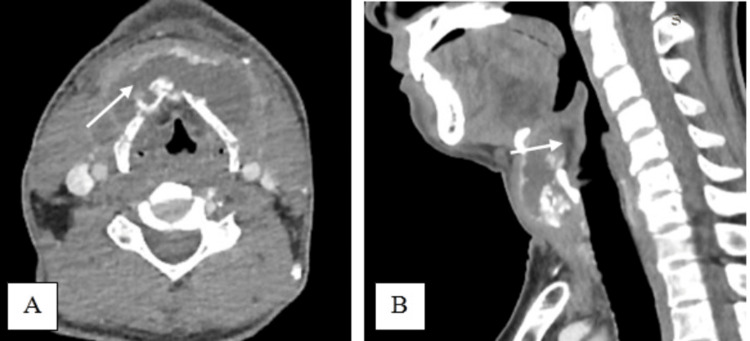
Axial view: Contrasted CT scan of the neck showing density fluid collection arising from perichondrium anterior to thyroid lamina with amorphous and multilobulated course calcification seen with erosion bilateral thyroid cartilage (white arrow) (A) Sagittal view: Contrasted CT scan of the neck showing collection extended into pre-epiglottic space (white arrow) (B).

**Figure 3:**
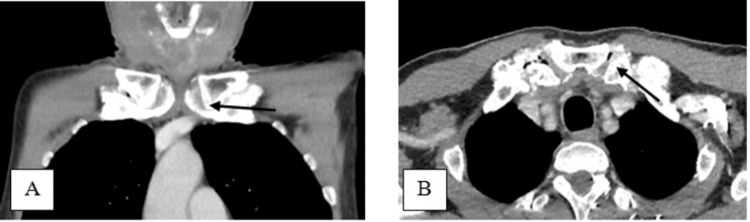
Coronal (A) and axial view (B) Contrasted CT scan of the neck showing periarticular calcification at the bilateral sternoclavicular joint (black arrow)

**Figure 4:**
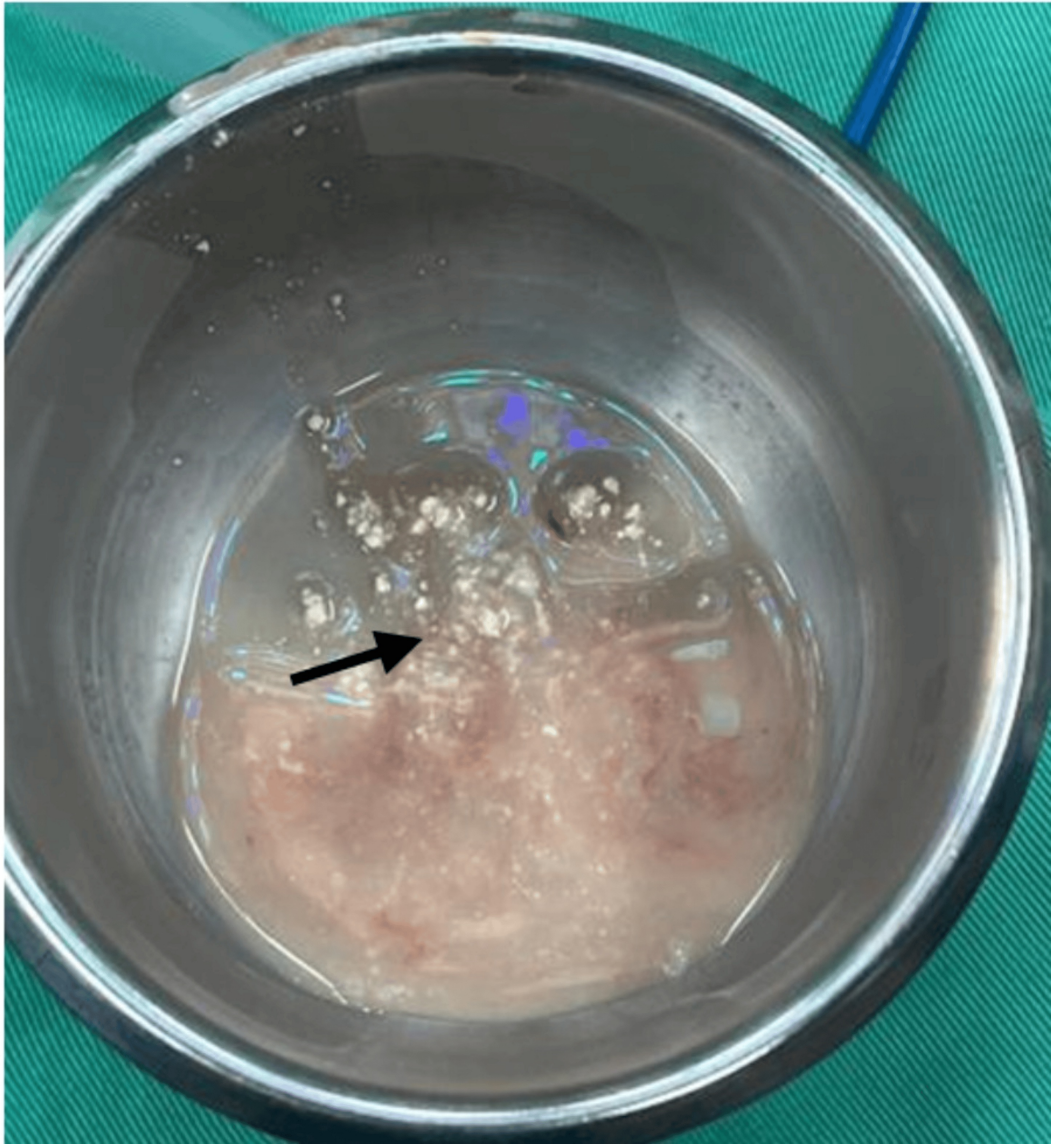
Thick pus with multiple tophi crystal (black arrow)

**Figure 5:**
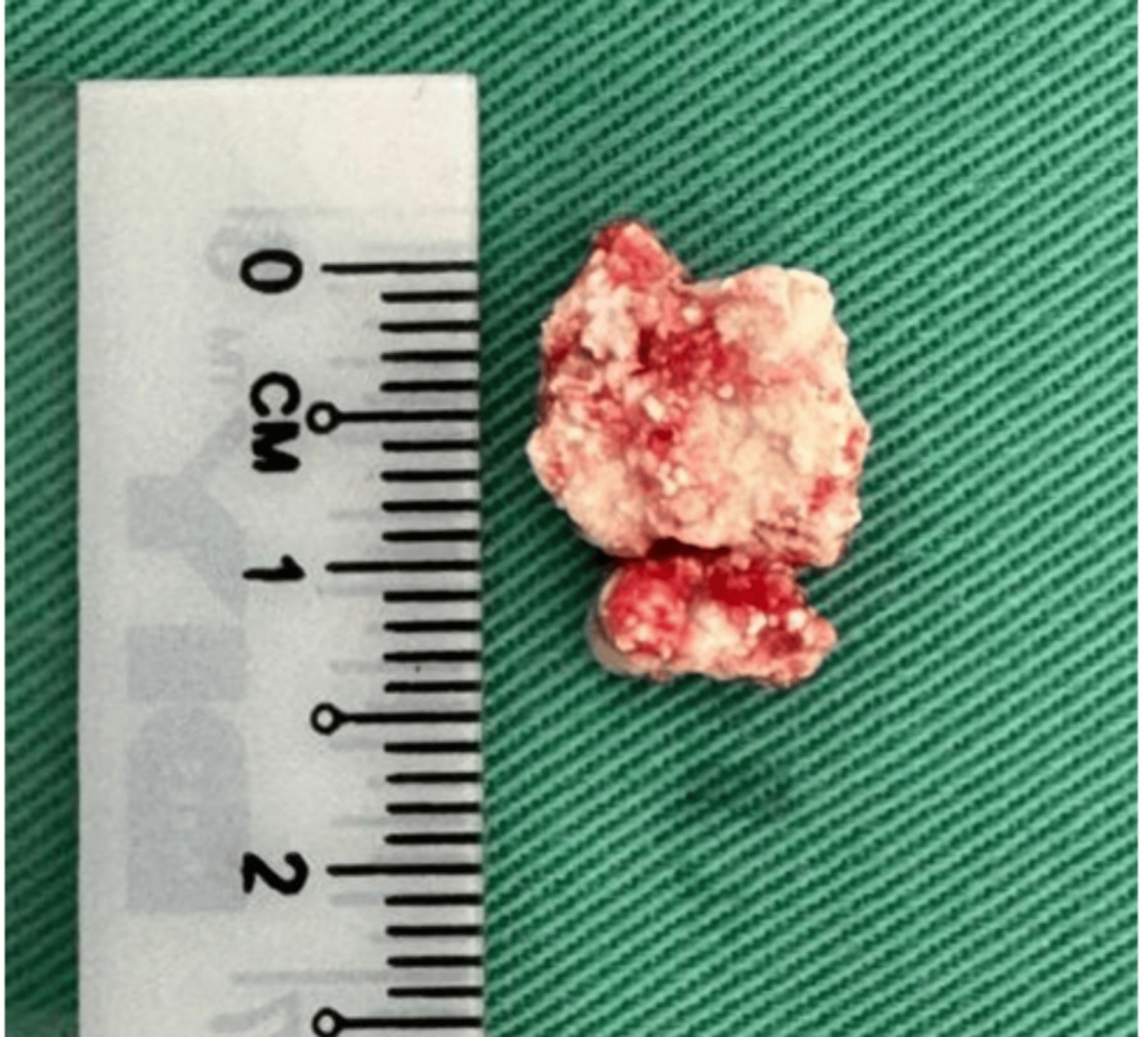
Tophy crystal measuring 1.4 cm

**Figure 6:**
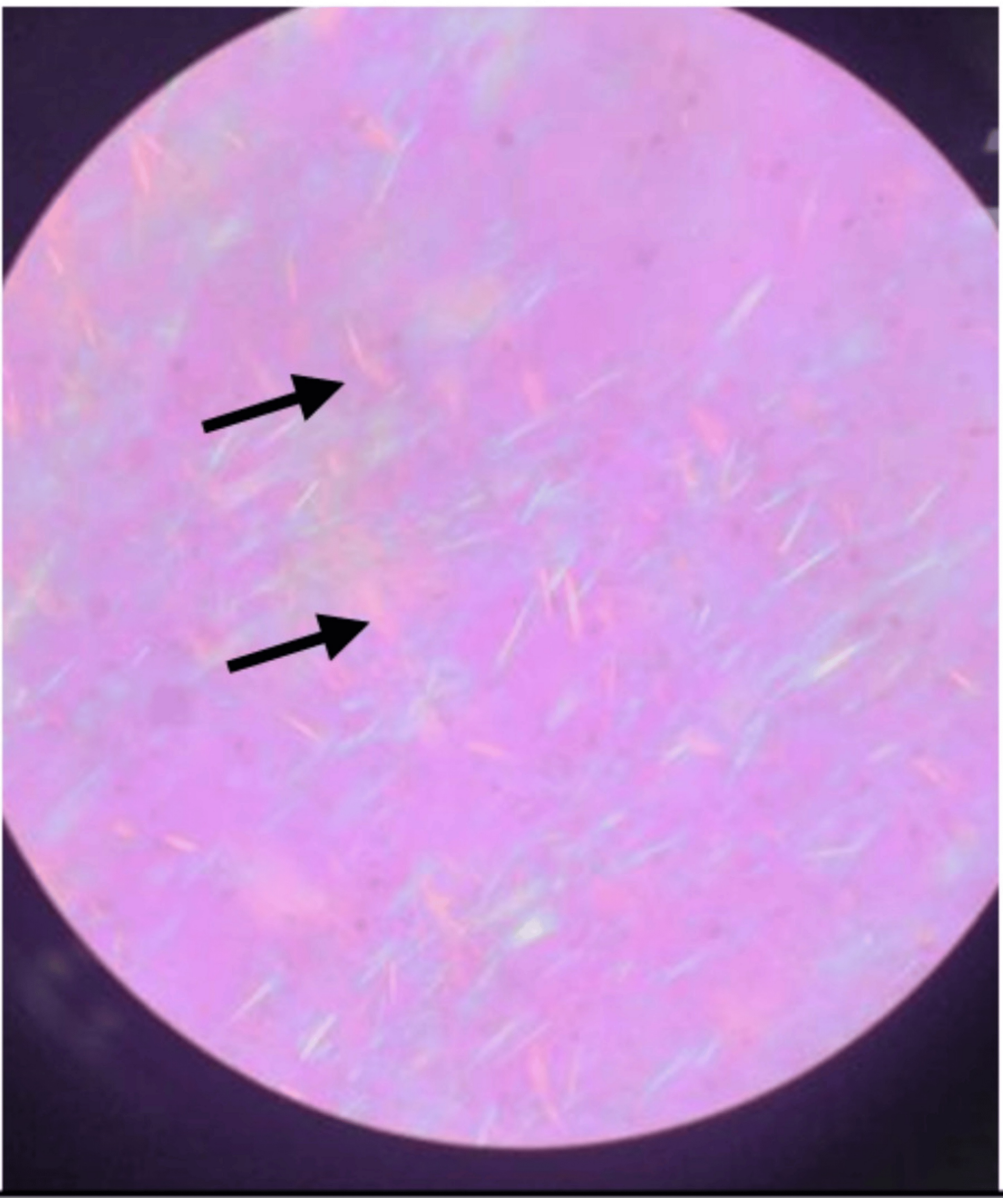
Examination under the microscope showing negative birefringent crystals (black arrow)

A 50-year-old male presented with a sudden onset of painless anterior neck swelling for two weeks with a rapid increase in size. Associated symptoms include dysphagia and change of voice, otherwise, there is no odynophagia, noisy breathing, fever, or trauma to the neck. He was under health care clinic follow-up for a long history of gouty arthritis. He was on a regular dose of tablet allopurinol 100 mg daily, and his serum uric acid level on admission was 4.57 mg/dl, which is within the normal range of 3.5 to 7.2 mg/dl. Clinically he was comfortable and afebrile during the consultation. A neck examination revealed a well-defined anterior neck swelling, measuring 6 cm × 7 cm at the level of the thyroid cartilage, which moved with deglutition. The swelling was soft, and non-tender with a globular surface and normal skin overlying confined to the thyroid cartilage area (Figure 1). Cervical lymph nodes were not palpable. Fiberoptic scope revealed mild edema at the arytenoid, aryepiglottic fold, and vallecular with pooling of saliva around the pyriform fossa bilaterally. Other laryngeal structures were normal and vocal cords were mobile. Ultrasound revealed a midline neck mass with intralesional calcification of the cartilaginous component and suspicious erosion of the thyroid cartilage. A contrasted CT scan of the neck revealed a fluid collection of 3.6 cm × 7.6 cm × 5.3 cm in size arising from subperichodrium of anterior thyroid lamina with amorphous and multilobulated coarse calcification seen with erosion of bilateral thyroid cartilage. The collection extends posteromedially into pre-epiglottic space (Figure 2). There is periarticular calcification at the bilateral sternoclavicular joint suggestive of gout (Figure 3). He underwent an incision and drainage under general anesthesia and drained 20 cc thick pus with tophi crystal (Figure 4). Pus culture and acid-fast bacilli yield negative results. The biggest tophy crystal measuring 1.4 cm × 1.4 cm was sent for histopathological examination (Figure 5). Microscopic examination revealed negative birefringent crystals confirming gout (Figure 6). The patient had an uneventful surgery and was discharged well with daily wound dressing at the nearest clinic. On follow-up, the wound has healed with no gouty tophus.

## Discussion

Gout is a prevalent long-term condition marked by ongoing disturbances in purine metabolism and decreased uric acid elimination. Gouty arthritis impacts just 15% to 25% of individuals with hyperuricemia; 95% of those affected are men, and the likelihood of onset appears in the fifth decade. Hyperuricemia is a significant cause of gout and provides a necessary metabolic basis for its development [1]. The human body’s synthesis and excretion of uric acid are in dynamic balance. When these processes are disturbed, blood uric acid levels increase, resulting in hyperuricemia [2].

High blood uric acid levels will cause urate crystals to precipitate that can lodge in different tissues inside or outside the joints forming small firm lumps called tophi. These tophi are not usually painful and can be classified into common sites or unusual sites [3]. The first metatarsophalangeal joint is the most often impacted, followed by the wrist, ankle, and knee joints [4]. In the head and neck, gouty tophi are regarded as unusual. It has been reported in the nose [5] and middle ear [6]. Laryngeal gout is extremely rare. Most reported cases occur at the vocal cord [7] and cricoarytenoid [1]. Only a few cases occur at the thyroid cartilage.

The laryngeal gout can present with anterior neck swelling and associated symptoms of dysphagia and hoarseness. When tophy impacts the vocal cords or subglottic area, it can lead to varying levels of hoarseness. Involvement of the cricoarytenoid joint is intensely painful, and in serious instances, it may result in respiratory distress [8]. Laryngeal gout occurring alongside tendonitis of the longus colli muscle can lead to intense dysphagia and throat discomfort [9]. The presence of tophi in the larynx and trachea can result in respiratory stenosis, causing respiratory obstruction [10]. Thus, if gouty tophi are suspected in the larynx, surgical intervention is needed to avoid complications such as upper airway obstruction.

The CT scan is the best modality that can provide differential diagnoses such as anterior neck abscess, tuberculosis, and pyogenic chondritis. The thyroid cartilage contains gouty tophi, which usually shows up on CT scans as rounded masses that are denser than soft tissue but less dense than calcifications [11-12]. Our patient presented with anterior neck swelling confined to the thyroid cartilage area which was non-tender and not inflamed. It was sudden in onset and had no history of trauma, not suggestive of neck abscess. Pus was aspirated in the clinic and sent for culture and acid-fast bacilli and the result was negative. A contrasted CT scan shows fluid density with amorphous calcification at the perichondrium of the thyroid cartilage with erosions suggesting infection concurrent involvement of the periarticular excessive ossification at the bilateral sternoclavicular joint suggestive laryngeal gout. The collection also extended into the posteromedially of pre-epiglottic space because of thyroid cartilage erosion.

Individuals with laryngeal gout involvement often possess a prolonged history of chronic polyarticular arthritis. Usually, serum urate levels for known cases of gout are more than 7 mg/dl for men and more than 6 mg/dl for women. However, it is possible that gout attacks can still happen even when serum urate levels are normal, with a prevalence ranging from 12% to 63.3%[13]. In this case, the patient had laryngeal gout even though he complied with treatment and had normal uric acid levels, which were around 4.57 mg/dl.

Although imaging tests can give for differential diagnosis, the definitive method for diagnosing gouty tophi continues to be pathological examination. The pathological features of gouty tophi consist of needle-like, negatively birefringent monosodium urate (MSU) crystals encircled by soft tissue and inflammatory cells visible under the microscope [14]. In this study, the microscopic examination of crystal tophi showed negative birefringent crystals which confirmed the diagnosis.

The treatment of laryngeal gout requires the implementation of an organized management strategy, with an emphasis on lifestyle changes as a primary approach. It includes prioritizing weight reduction, abstaining from alcohol consumption, and avoiding foods high in purine. However, in certain cases, they may need to proceed with surgical intervention if the patient is having obstructive symptoms. In this case, the diagnosis is still not yet established, and the patient has obstructive symptoms. Therefore, to prevent respiratory distress, surgical incision and drainage are required. Post-operative treatment options may involve the use of nonsteroidal anti-inflammatory drugs or steroids. To avoid reoccurrence post-surgery, the patient was directed to maintain oral administration of febuxostat and colchicine to reduce uric acid.

By presenting this case and examining existing literature, we aim to offer valuable insights for further study, enabling healthcare professionals to recognize the atypical symptoms and unusual distribution of gout, enhancing diagnostic accuracy, and providing optimal treatment options for patients.

## Conclusions

This case emphasizes the need to maintain a degree of suspicion of laryngeal gout in patients with anterior neck swelling and a known history of gout. A CT scan can help determine and provide a differential diagnosis. Microscopic examination of samples is required to establish a diagnosis. Normal serum uric acid levels cannot prevent laryngeal gout from occurring. A proper sample of the tophus crystal should be collected, accompanied by suitable medical treatment and lifestyle changes to achieve purine metabolic equilibrium. Heightened awareness and reduced suspicion thresholds could enhance clinical identification and enable earlier diagnosis and management of laryngeal gouty tophus.
